# Gray matter imaging in multiple sclerosis: what have we learned?

**DOI:** 10.1186/1471-2377-11-153

**Published:** 2011-12-12

**Authors:** Hanneke E Hulst, Jeroen JG Geurts

**Affiliations:** 1 Department of Radiology, VU University Medical Centre, PO Box 7057, 1007 MB, Amsterdam, The Netherlands; 2 Department of Anatomy and Neurosciences, section of Clinical Neuroscience, VU University Medical Centre, Van der Boechorststraat 7, 1081 BT Amsterdam, The Netherlands

## Abstract

At the early onset of the 20^th ^century, several studies already reported that the gray matter was implicated in the histopathology of multiple sclerosis (MS). However, as white matter pathology long received predominant attention in this disease, and histological staining techniques for detecting myelin in the gray matter were suboptimal, it was not until the beginning of the 21^st ^century that the true extent and importance of gray matter pathology in MS was finally recognized. Gray matter damage was shown to be frequent and extensive, and more pronounced in the progressive disease phases. Several studies subsequently demonstrated that the histopathology of gray matter lesions differs from that of white matter lesions. Unfortunately, imaging of pathology in gray matter structures proved to be difficult, especially when using conventional magnetic resonance imaging (MRI) techniques. However, with the recent introduction of several more advanced MRI techniques, the detection of cortical and subcortical damage in MS has considerably improved. This has important consequences for studying the clinical correlates of gray matter damage. In this review, we provide an overview of what has been learned about imaging of gray matter damage in MS, and offer a brief perspective with regards to future developments in this field.

## Background

For many years, focal inflammatory demyelination in the white matter (WM) was considered the most important pathological 'hallmark' of multiple sclerosis (MS). However, demyelination in the cerebral cortex was already observed in early pathology studies by Sander (1898), Dinkler (1904), Schob (1907) and Dawson (1916) [1-4]. After these initial, largely casuistic, descriptions of demyelination in the gray matter (GM) of MS patients, the topic was largely disregarded. This was mostly due to difficulties involved with the visualization of cortical GM lesions in the post mortem setting, in which conventional histochemical staining procedures were applied, as well as to a predominant attention for the generally more conspicuous process of inflammatory WM demyelination.

**Figure 1:**
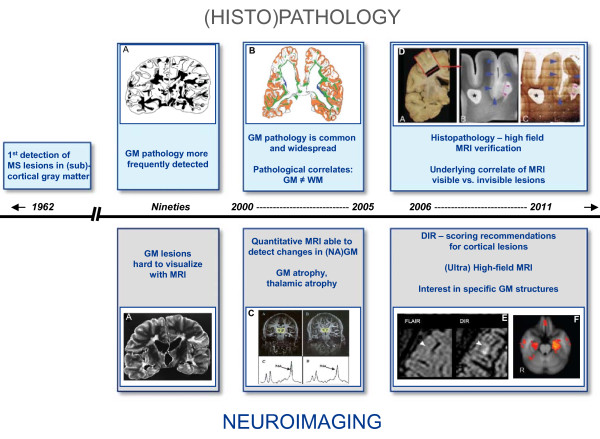
**Timeline of GM imaging in MS**. A schematic overview of developments in the field of GM imaging in MS from the beginning of the 20^th ^century until now. **A, B, D**) Reproduced from Kidd et al. [7], Kutzelnigg et al. [19], and Schmierer et al. [88] respectively, all with permission from Oxford University Press. **C**) Reproduced from Cifelli et al. [100] with permission from John Wiley and Sons. **E, F**) Reproduced from Geurts et al. [69] and Roosendaal et al. [126] respectively, both with permission of the Radiological Society of North America.

However, by the start of the 21^st ^century, the focus within MS research slowly shifted back from WM to GM. In 2003, when new immunohistochemical staining techniques that improved the ex vivo detection of GM damage had become available, the presence and extent of GM demyelination was described in detail and pathophysiological processes causing GM damage, as well as its visualization with modern magnetic resonance imaging (MRI) techniques, became central issues in MS research (see Figure 1).

This review will focus on what has been learned in the past decade of imaging GM pathology in MS. As will be shown, visualization of GM demyelination was difficult at first, but improved upon technical developments in the field. An important question that now remains is whether MRI visualization of GM pathology in MS is sufficient, or whether further improvement is still needed.

### GM involvement in a WM disease

After the first pivotal reports of GM damage in MS in the early 20th century, it was not until 1962 that Brownell and Hughes reported that 26% of the macroscopically visible lesions found in their post mortem material of 22 MS patients were (partly) located in or around the cortical and subcortical GM [5]. Extensive involvement of the cortex in MS patients was later confirmed in histopathology [6] and in a study combining post mortem MRI and conventional histology [7].

At that point in time, the detection of GM demyelination in tissue sections was difficult, as standard histochemical myelin stains like luxol fast blue (LFB) were not sufficiently adequate for visualization of changes in GM myelin density. Detection of GM pathology with conventional T2-weighted MRI also proved to be difficult because of the intrinsically low myelin density in the cortex, which generates little contrast between normal GM and demyelinated GM lesions, and because of the fact that cortical lesions may be small and partial volume effects with cerebrospinal fluid in the sulci can interfere with reliable lesion detection [7,8]. To improve the detection of cortical lesions on MRI, newer techniques like fast fluid-attenuated inversion recovery (FLAIR) MR sequence were used [9,10]. FLAIR improved lesion detection in the cortex and in (juxta)cortical areas as compared to conventional T2-weighted MRI [9-14]. However, the reported prevalence of cortical lesions on MRI was still much lower than that reported in post mortem studies [10].

### Description and classification of GM pathology in MS

**Figure 2:**
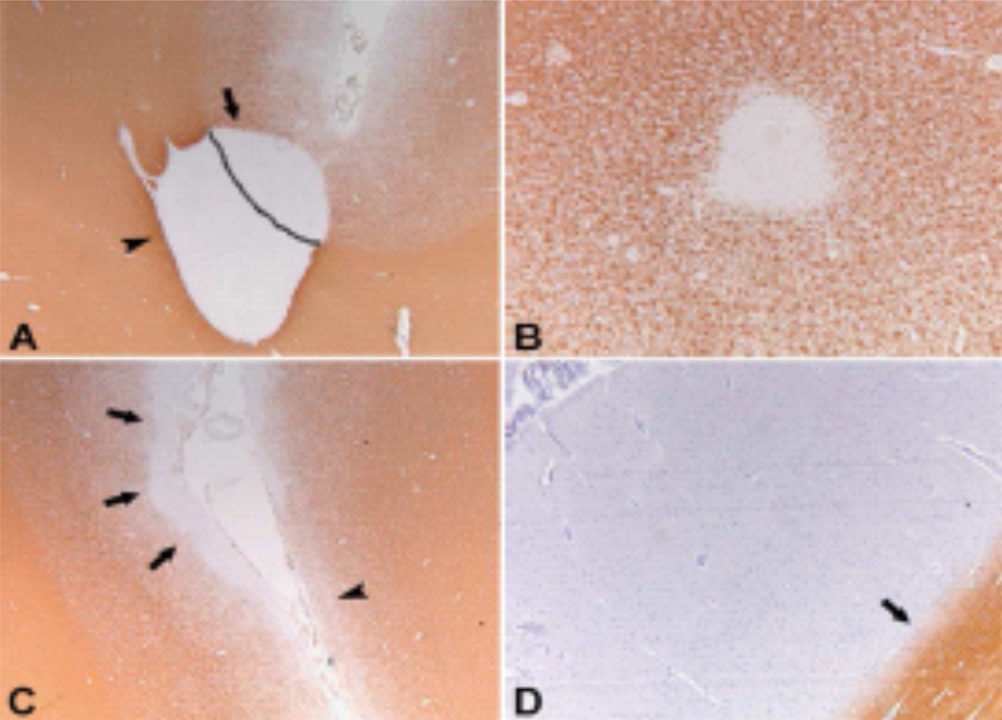
**Pathological classification system of GM lesions in MS**. GM lesion classification system as proposed by Bo et al,. 2003. Type 1 lesions (**A**) extend through both white and gray matter. Type 2 lesions (**B**) are intracortical, having no contact with white matter or with the surface of the brain. Type 3 lesions (**C**) extend inward from the surface of the brain. Type 4 lesions (**D**) extend through the whole width of the cortex without reaching into white matter. Reproduced from Geurts and Barkhof [131] with permission from Elsevier.

Histopathological detection of intracortical MS lesions improved with the use of myelin protein immunohistochemistry in the beginning of the 21^st ^century. Using immunohistochemistry for myelin basic protein (MBP) and proteolipid protein (PLP), Bö and colleagues shed more definitive light on the extent and distribution of cortical demyelination in MS [15,16]. In chronic MS cases myelin loss was found in 26.5% of their systematically examined areas of cerebral cortex [15]. The investigators proposed a classification system for cortical lesions which distinguished four different cortical lesion types: the mixed GM-WM (type I) lesions and the purely cortical (type II, III, and IV) lesions [16] (see Figure 2).

The pathology of GM lesions generally differs considerably from that of WM lesions. Lymphocyte infiltration, complement deposition, and blood-brain-barrier disruption have not been detected in GM lesions, whereas WM lesions are known to harvest substantial inflammation [16-18]. Another pivotal study by Kutzelnigg and colleagues showed demyelination of the MS cortex and reported extreme cases with more than 70% of cortical demyelination [19]. Besides demyelination in the cerebral cortex, demyelination was later also found in the cerebellum [20], in deep gray matter structures [21,22], and in the GM of the spinal cord [23].

Pathological studies demonstrated that cortical demyelination is most frequent and extensive in patients with a longer disease duration (secondary progressive MS; SPMS) and with a more progressive form of MS (primary progressive MS, PPMS), whereas it was found to be rare in relapsing remitting MS patients (RRMS) [19,21]. However, GM demyelination was recently found in biopsy material of an early MS patient, even before disseminated WM lesions on MRI became visible [24].

Although histopathological studies had improved as a result of the new staining methods, GM lesions remained extremely difficult to detect on conventional MRI. For example, it was shown that T2-weighted and FLAIR imaging detected only 3 to 5% of a total of 63 histopathologically defined cortical lesions [25]. Nevertheless, the (juxta)cortical lesions that were detected with these MRI sequences were clearly associated with cognitive impairment, epilepsy, depression, fatigue, and physical disability in MS patients [26-32].

In parallel, MRI studies focused on imaging the normal appearing GM of MS patients (NAGM; i.e., GM that looks normal on conventional T2-weighted MRI, but might nevertheless be histopathologically abnormal) using more advanced, quantitative MRI techniques. These techniques are generally more sensitive to (subtle) pathology in the GM than conventional MRI sequences [33]. Clinically relevant abnormalities in the NAGM of MS patients were reported with magnetization transfer imaging (MTI) [34-37], T1-relaxometry [38,39], diffusion tensor imaging (DTI) [40,41], and proton magnetic resonance spectroscopy (MRS) [42-44] in both cortical and subcortical GM areas in MS. These measured abnormalities most likely reflect subtle tissue changes due to lesions or independent from lesions.

To sum up, around the turn of the century, GM pathology in MS became increasingly recognized as an important pathological hallmark in this 'typical' WM disease. The introduction of new histopathological staining methods improved the visualization of GM damage and revealed that GM demyelination was frequent, widespread, and more extensive in patients with longer disease duration. The pathological substrate of GM lesions was found to be different from WM lesions, as GM lesions are largely non-inflammatory. On conventional MRI, only a minority of GM lesions could initially be visualized. However, these lesions were associated with clinical deterioration. Besides cortical lesions, quantitative MRI also demonstrated clinically relevant abnormalities in the NAGM of MS patients.

### GM atrophy and cortical thinning

Whereas the detection of focal GM lesions was difficult using conventional MRI measures, GM atrophy measurements proved to be robust and reliable using standard MRI sequences [33]. Automated methods to estimate brain volume were reproducible, both within and between research centres [45,46]. Decreased GM tissue volume, as well as cortical thinning, were found in MS patients compared to healthy age-matched controls and were observed already early in the disease course and across different MS types [47-59]. In a longitudinal study it was shown that whole brain atrophy rates were similar over time in stable Clinically Isolated Syndrome (CIS) patients, but steadily increasing as diseased severity increased (RRMS and SPMS) [60]. Interestingly, a significant and disproportionate increase in GM atrophy occurs in MS patients in more advanced disease stages [55,61], while WM atrophy rates accumulate more constantly over time [60]. GM atrophy and cortical thinning were significantly associated with physical disability and cognitive decline [47-50,55,56,62-66], and importantly, measures of GM atrophy showed stronger correlations with clinical parameters than WM damage [56,60,61,66].

### New imaging techniques to detect cortical GM lesions

In 1994, a new MRI sequence, the double inversion recovery (DIR), was introduced. This technique provided excellent distinction between the cerebral cortex and the WM in the healthy human subjects by suppression of the signal from WM and the cerebrospinal fluid (CSF) [67]. DIR proved to be superior in comparison to FLAIR where it concerned intratentorial lesions and lesions with low contrast on T2-weighted MR sequences [68]. However, it was not until several years later that DIR was applied to image GM pathology in MS [69]. In 2005, MR imaging with a 3D DIR sequence demonstrated increased intracortical lesion detection in the MS brain, as well as improved distinction between juxtacortical and mixed WM-GM lesions. A relative gain of 152% of DIR over FLAIR was described in the detection of cortical lesions [69]. Using DIR at a higher field strength (3T) led to an even further increase of detected cortical lesions in MS patients [70].

A series of important scientific contributions by Calabrese and colleagues subsequently confirmed histopathology studies by reporting that intracortical lesions detected on DIR are already present early in the disease [71-74] and are found in both relapse-onset and PPMS patients [74,75]. The investigators further showed that cortical lesions are most frequently found in SPMS patients, in patients of the male sex, and in patients who also had IgG oligoclonal bands in the CSF [76,77]. A relative sparing of the cortex was found for patients with benign MS [72]. The presence of cortical lesions was associated with clinical disability, brain volume loss and/or cognitive impairment [73,75-79]. Longitudinal DIR studies showed an increase in cortical lesion number and/or an increase in lesion size over time [73,75,77,80].

These results notwithstanding, the use of different DIR sequences and different lesion scoring criteria in different research centres made the comparison of available cortical lesion data in the literature challenging. Therefore, in an attempt to improve consistency of cortical lesion scoring between raters and centres using DIR, international cortical lesion scoring guidelines were proposed [81,82]. These DIR scoring guidelines were subsequently verified in the post mortem setting and showed that although DIR is highly pathologically specific (90% of cortical hyperintensities identified related to actual lesions in post mortem tissue), the sensitivity of DIR is rather poor [82]. Especially subpial cortical lesions were found to be difficult to detect on DIR.

So far, no specific pathological properties were found that determine whether cortical lesions become MRI-visible or MRI-invisible. Instead, it was shown that the visibility of cortical lesions on MRI is predominantly determined by their size. Furthermore, MRI-visible cortical lesions were strongly associated with a higher total cortical lesion load in brain tissue specimens, which suggests that when cortical lesions become visible on MRI, they merely reflect 'the tip of the pathological iceberg' [83].

Other studies aiming to improve visualization of cortical GM lesions used phase-sensitive inversion recovery (PSIR) and 3D T1 weighted sequences [84,85]. Future post mortem work will have to show whether sensitivity and specificity for cortical GM lesions may further improve when combinations of T1-based techniques, PSIR and DIR are used, when compared to DIR alone. Ideally, this would lead to a recommendation as to which (combination of) technique(s) should be used to optimally visualize (cortical) GM pathology in vivo, and further increase our understanding about the role of GM damage in clinicocognitive deterioration in MS.

### High-field and ultrahigh-field MRI

With the introduction of (ultra) high-field MRI, signal-to-noise ratio, spatial resolution, and image contrast improved, which all benefited the detection of GM lesions [86-88]. In vivo studies using 2D T2*-weighted gradient-echo and 3D T1-weighted magnetization-prepared rapid-acquisition GRE sequences at 7T or higher were able to provide high-resolution anatomical images of cortical lesions [89-91]. It was recommended that a combination of MR sequences be used at higher field strength, as different techniques may provide complementary information in the definition of cortical lesions [92]. Furthermore, and this is new when compared to standard field, it was shown that ultra high-field MRI enables accurate visualization of subpial GM lesions [15,88,91].

### Non-neocortical GM damage

**Figure 3:**
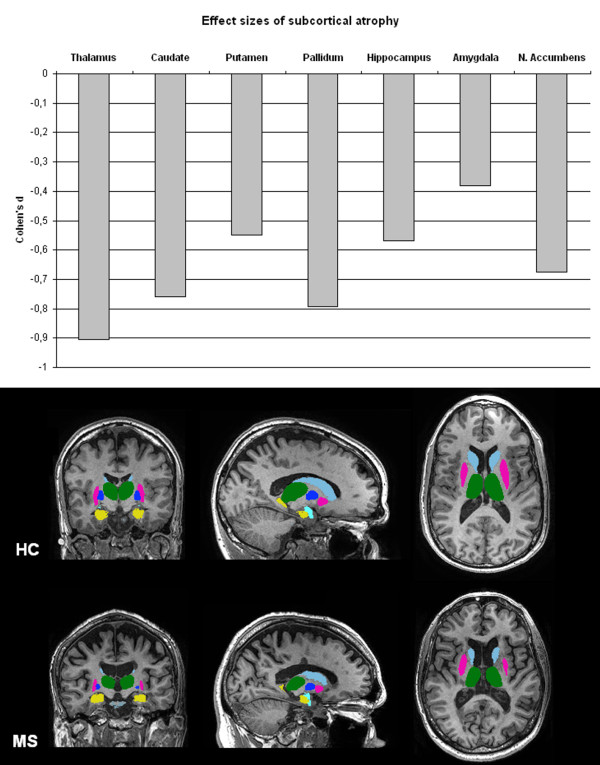
**Subcortical GM damage in MS**. Subcortical atrophy, measured using FIRST (part of FSL 4.1: http://www.fmrib.ox.ac.uk/fsl/). Above: Effect sizes of subcortical atrophy in a cohort of 120 early RRMS patients, six years post-diagnosis. Below: Two examples of segmented subcortical structures in a healthy control (HC, above) and an age-matched RRMS patient (MS, below).

MRI and histopathology studies showed that atrophy and demyelinated lesions do not only exist in the cortical GM but also in other GM structures such as the thalamus, hippocampus, caudaute, putamen, pallidum, claustrum, hypothalamus, amygdala and substantia nigra [21-23,42,93-105], see Figure 3. Hypothalamic lesions were found to be numerous in a post mortem MS dataset [96] and higher inflammatory activity of hypothalamic lesions was associated with a worse disease course [97].

Of all the GM structures, the thalamus has been studied most extensively with MRI [21,22,42,99-110]. One of the reasons that the thalamus received broad attention is because of the extensive reciprocal connections with the cortex and subcortical structures which makes this brain structure particularly sensitive to pathological changes in other areas of the brain [100]. Several studies focused on the volume of non-neocortical GM structures and showed thalamic atrophy in all different MS disease types [102-104,106-109,111-113] (see Figure 3). In CIS patients, thalamic atrophy was already present [103,104,108,112], and interestingly a reduction in thalamic fraction was more pronounced in RRMS patients compared to SPMS patients [104]. Additionally, in a longitudinal study thalamic atrophy was found to be more pronounced in RRMS patients compared to regional cortical atrophy [111]. This indicates that neuronal damage in the thalamus occurs early in the disease course. In terms of clinical relevance, thalamic atrophy has been associated with disability, cognitive impairment, and fatigue [99,104,107,110]. Furthermore, thalamic atrophy was seen in paediatric MS patients and PPMS patients as well [109,113,114]. Using quantitative MR techniques, changes in the chemical composition of thalamic tissue could be detected. A reduction of N-acetylaspartate (NAA) in the thalamus was found with MRS and is thought to reflect neuro-axonal loss [42,100,102]. MTI showed a reduced magnetization transfer ratio (MTR) in the thalamus, an abnormality which was already found within the first five years of the disease [101,104]. And also changes in thalamic DTI measures were partly associated with disability, brain volume, and lesion load [105].

The hippocampus was found to be a predilection site for GM demyelination in MS [21,95,115]. In vivo, areas of signal abnormality in the hippocampus were also identified by using DIR [116], as well as increased levels of myo-inositol using MRS [42]. In addition, hippocampal atrophy was found in RRMS, SPMS, and PPMS patients, imaged at 1.5T and 3T [117,118]. At 3T, atrophy was reported to start in the cornu ammonis 1 (CA1) region of the hippocampus in RRMS, only to expand to other CA regions in more advanced stages of the disease (SPMS) [118].

**Figure 4:**
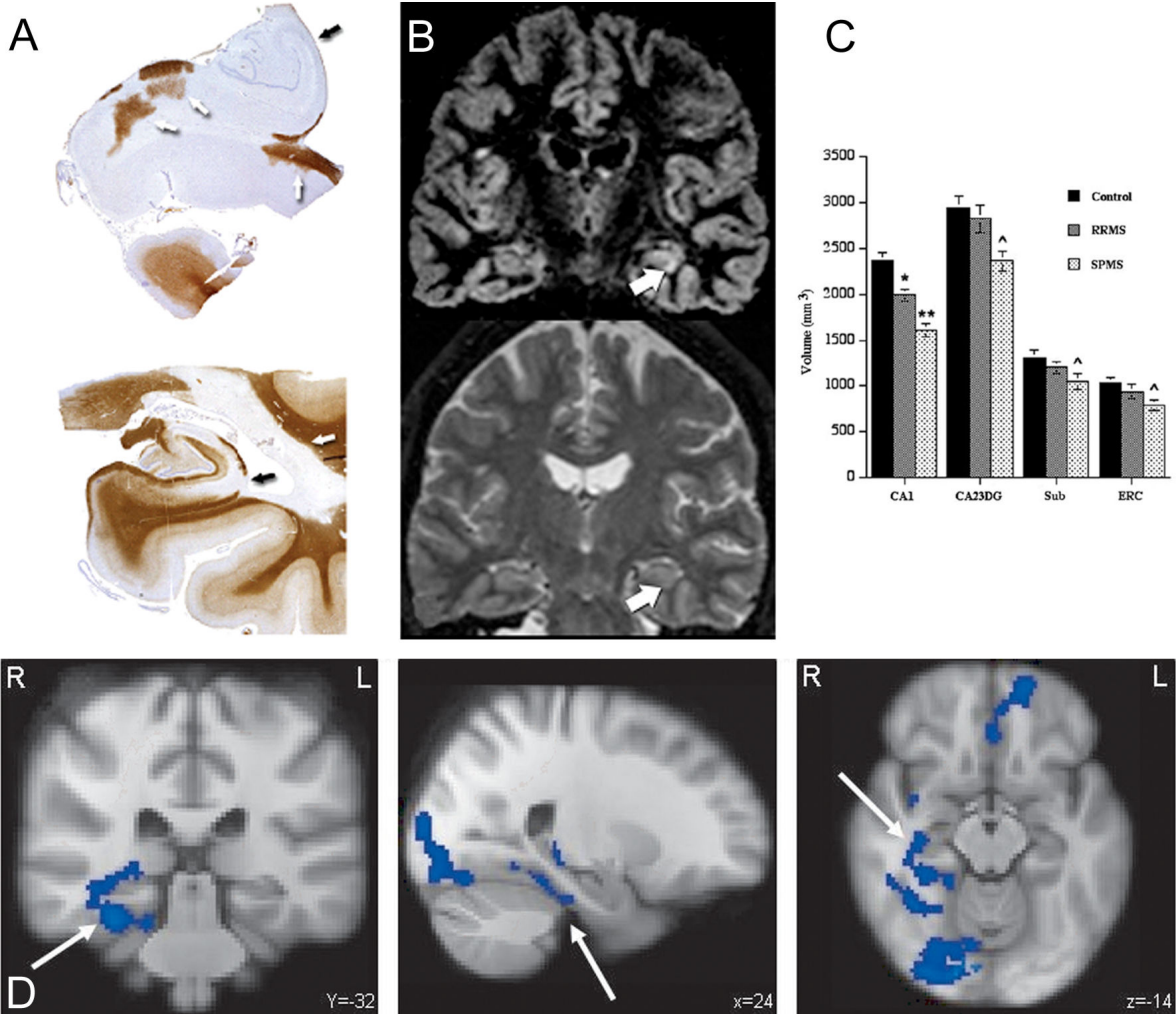
**Non-neocortical GM damage: the hippocampus**. **A) **Extensive hippocampal demyelination observed in post-mortem material. Reproduced from Geurts et al. [95] with permission from the American Association of Neuropathologists Inc. **B) **In vivo detection of hippocampal lesions using DIR imaging. Reproduced from Roosendaal et al. [116] with permission from John Wiley and Sons. **C) **Hippocampal atrophy on high field MRI provides detailed information on the specific location in the hippocampus where atrophy is present. Reproduced from Sicotte et al. [118] with permission from Oxford University Press. **D) **Anno 2011 it is besides structural changes also possible to study functional changes in the hippocampus; the blue areas indicates reduced hippocampal activity in cognitively impaired MS patients compared to healthy controls. Reproduced from Hulst et al. [127] with permission from John Wiley and Sons.

Besides structural differences in the MS brain, functional changes can be studied as well by using e.g. functional MRI (fMRI). Different cognitive paradigms for task-fMRI have been used so far, as well as resting-state fMRI. Several differences between healthy controls and MS patients were found in terms of brain activation and brain connectivity [119-125]. Some fMRI studies focused on a particular GM brain structure or functional anatomical system rather than on a specific cognitive domain. For example, reduced functional connectivity was reported between the hippocampus and its anatomical input c.q. target areas, including the anterior cingulate gyrus, thalamus, and prefrontal cortex. These changes were more pronounced in MS patients with hippocampal atrophy, but were also detected in patients without hippocampal volume loss [126]. Additionally, in a task-specific fMRI study, differences in hippocampal function during a memory encoding task were detected between MS patients with and MS patients without cognitive decline. These findings were independent of differences in hippocampal volume and number of hippocampal lesions [127] (see Figure 4).

To summarize, during the past few years, the number of studies investigating GM damage in MS has tremendously increased. GM atrophy and cortical thinning were shown to be robust and reliable measures. GM atrophy correlated more strongly than WM atrophy with disability and cognitive impairment. New imaging techniques, such as DIR, improved the detection of cortical lesions in vivo. With the introduction of (ultra) high-field MRI, the visualization of cortical lesions improved even further. Recently published international consensus guidelines for scoring cortical lesions on DIR will likely increase the consistency between different research groups, and will improve the comparability of different DIR studies. Post mortem work demonstrated that DIR is pathologically specific, but lacks sensitivity. This means that many cortical lesions are still missed and MRI detects only 'the tip of the pathological iceberg'. Despite their under representation on MRI, cortical lesions were shown to be clinically relevant. Both imaging and histopathology studies showed that GM damage in MS is not limited to the neocortex; the cerebellum, spinal cord, and non-neocortical GM structures are affected as well.

### A brief future perspective of GM imaging in MS

From a pathological point of view, it is clear that GM damage is common, widespread, and different from WM damage. A challenging next step is to investigate the cause(s) of GM pathology. For example, whether damage to the GM develops purely as a result of inflammatory demyelination in the WM or whether pathological processes in these two compartments of the brain are less connected than formerly thought, still remains to be elucidated [128-130].

As discussed in this review, from the neuroimaging perspective, MRI techniques have substantially improved over the past years and several new imaging modalities to better detect GM abnormalities are now available. It is possible to visualize cortical lesions with DIR, T1-based or phase-sensitive sequences, as well as with (ultra) high-field MRI. Moreover, GM tissue loss can be reliably measured with the help of conventional MR sequences, and more subtle tissue changes can be observed with quantitative MRI. Finally, the function of specific GM structures or functional systems, as well as functional and structural connectivity changes within the brain can be imaged with MRI. With all these different imaging techniques to visualize GM pathology in vivo, it now remains to be determined which image modality, or combination of imaging modalities, best explains or predicts clinical symptomatology, and whether these techniques can be reliably used to monitor future treatment effects. Furthermore, the spatiotemporal relation between structural and functional changes, as well as changes in network dynamics in the brain as a consequence of disease will be difficult but exciting topics for future research in this field. The past few years of GM imaging research have seen tremendous progress; with the knowledge and technology now available, it is likely that ongoing joint efforts between different fields of research will create an even greater understanding of the role of GM pathology in MS in the near future.

## Conclusions

### What have we learned in GM imaging in MS?

▪ GM damage in MS is common and widespread, especially in chronic MS;

▪ The underlying pathological correlates of GM damage in MS are different from WM damage;

▪ GM pathology is present in all stages of the disease, but is more prominent in SPMS and PPMS compared to RRMS;

▪ Although a relatively non-specific measure of overall pathology, GM atrophy measurements are reliable and robust and correlate strongly with disability and cognitive impairment (more so than WM atrophy);

▪ Cortical lesions are of clinical relevance and are associated with a worse physical and cognitive performance;

▪ Cortical lesions have been difficult to visualize with conventional MRI, but due to newer imaging techniques (like DIR) lesion detection improved;

▪ Post mortem DIR verification showed high specificity of DIR lesions and increased sensitivity compared to conventional MR sequences; however, a substantial number of cortical lesions is still missed with in vivo MRI;

▪ International consensus guidelines on DIR cortical lesion scoring will likely improve the comparability of different DIR studies;

▪ (Ultra) high-field MRI holds promise for more precise detection of cortical lesions, with maybe even the possibility to categorize GM lesions in vivo according to the pathological classification system used post mortem; especially the detection of subpial lesions (type III lesions) is likely to benefit from the use of (ultra) high-field MR imaging;

▪ Non-neocortical GM damage is frequently detected in histopathological studies as well as on MRI. Thalamic and hippocampal abnormalities have been studied most extensively and were shown to correlate with clinical parameters;

▪ Besides *structural *changes in the GM of MS patients, *functional *changes can be detected as well by using fMRI; this should shed more light on the relationship between functional and structural changes in the MS brain in future studies.

## Competing interests

The authors declare that they have no competing interests.

## Authors' contributions

HEH and JJGG participated in the preparation of the manuscript. All authors read and approved the final manuscript.

## Pre-publication history

The pre-publication history for this paper can be accessed here:

http://www.biomedcentral.com/1471-2377/11/153/prepub
